# Ejection Fraction Improvement Following Contemporary High-Risk Percutaneous Coronary Intervention: RESTORE EF Study Results

**DOI:** 10.1016/j.jscai.2022.100350

**Published:** 2022-08-13

**Authors:** Jason Wollmuth, Mitul P. Patel, Thom Dahle, Aditya Bharadwaj, Thomas E. Waggoner, Jeffrey W. Chambers, Ernesto Ruiz-Rodriguez, Ehtisham Mahmud, Craig Thompson, D. Lynn Morris, RESTORE EF Investigators

**Affiliations:** a Providence Heart and Vascular Institute, Portland, Oregon; b Division of Cardiovascular Medicine, UC San Diego Health System, La Jolla, California; c Centracare Heart & Vascular Center, St. Cloud, Minnesota; d Division of Cardiology, Loma Linda University Medical Center, Loma Linda, California; e Pima Heart and Vascular, Tucson, Arizona; f Metropolitan Heart & Vascular Institute, Minneapolis, Minnesota; g Baptist Health Heart Institute/Arkansas Cardiology Clinic-Little Rock, Little Rock, Arkansas; h Division of Cardiology, Department of Medicine, NYU Langone Health, New York, New York; i Division of Cardiology, East Carolina Heart Institute at ECU, Greenville, North Carolina

**Keywords:** complete revascularization, high-risk percutaneous coronary intervention, Impella, left ventricular ejection fraction, percutaneous coronary intervention

## Abstract

**Background:**

Despite many reports of clinical outcomes in patients undergoing high-risk percutaneous coronary intervention (HRPCI) with hemodynamic support, little is known about whether this approach improves left ventricular ejection fraction (LVEF). The purpose of the present observational study was to examine, in an ideal patient population with Impella-supported HRPCI, whether there is an impact on left ventricular function at midterm follow-up.

**Methods:**

RESTORE EF is a multicenter, retrospective analysis of a prospectively collected observational data set that aimed to assess 90-day LVEF in patients undergoing Impella-supported nonemergent HRPCI (NCT04648306), who survived with no intervening cardiac procedures prior to the primary endpoint follow-up window (90-day LVEF assessment). Secondary endpoints included change in New York Heart Association Functional Classification and Canadian Cardiovascular Society Angina Grade at the last follow-up.

**Results:**

From August 2019 to May 2021, 406 patients were enrolled at 22 US sites. Age was 70.2 ​​± ​​11.4 ​​years; 26% were female. In paired assessment at 90-day follow-up, baseline LVEF improved from 35 ​​± ​​15% to 45 ​​± ​​14% (*N* = 251, *P* < .0001), with significantly greater improvement in patients with residual SYNTAX score I of 0. Percentage classified as New York Heart Association class III/IV decreased from 62% at baseline to 15% at last follow-up (*P* < .001), and percentage with Canadian Cardiovascular Society grade III/IV symptoms decreased from 72% to 2% (*P* < .0001).

**Conclusions:**

In an ideal cohort of HRPCI patients, there is a signal that hemodynamically supported HRPCI affords significant improvement in 90-day LVEF, with complete revascularization associated with greater LVEF improvement. These hypothesis-generating findings merit further assessment in large, all-comer studies and randomized trials.

## Highlights

• A significant LVEF improvement is noted in HRPCI patients who survive to 90 ​​days.
• HRPCI patients with complete revascularization show the highest improvement in LVEF.
• Significant heart failure and angina improvement is observed in normal baseline LVEF patients.
• This is the largest study to date looking at LVEF changes 30 ​​days after PCI.

## Central Illustration

**Figure:**
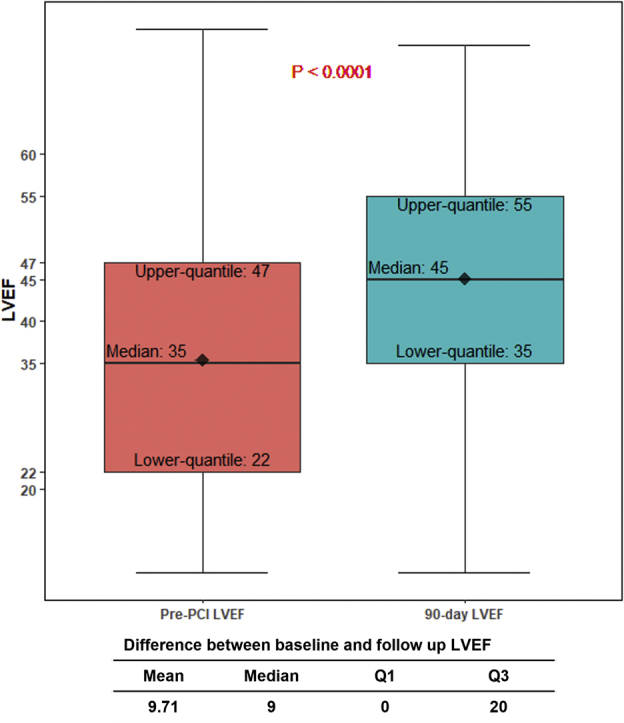

## Introduction

Patients with highly complex coronary disease, often turned down for coronary artery bypass surgery (CABG) due to concomitant risk factors such as significant comorbidity burden, patient frailty, or prior CABG, can, in some cases, be treated with high-risk percutaneous coronary intervention (HRPCI), using percutaneous mechanical circulatory support to prevent hemodynamic deterioration during PCI. Prophylactic hemodynamic support options include a percutaneous left ventricular (LV) assist device (the Impella microaxial rotary pump), which originally received Food and Drug Administration approval as temporary support for HRPCI procedures following performance in the PROTECT II randomized controlled trial (RCT). A depressed LV ejection fraction (LVEF) ​​<35% was 1 of the trial inclusion criteria, as it is understood that impaired LV function poses challenges to multivessel revascularization due to active hemodynamic derangements at rest, with general intolerance of added, although transient, ischemic insults.

However, since the PROTECT II RCT, a large Food and Drug Administration-audited postapproval study with clinical events committee adjudication has been performed, which resulted in broadening of the HRPCI indication to include patients without depressed LVEF for whom HRPCI is deemed the appropriate therapeutic option, per heart team decision. These patients may be deemed at risk of hemodynamic collapse during the procedure due to highly complex, high-risk coronary occlusions and/or a combination of other factors such as patient age, frailty, and comorbid conditions—particularly when pursuing complete revascularization.

The Impella 2.5 and CP (Abiomed) are currently approved for this indication under premarket approval in the United States and CE mark in Europe and other countries.^1^^,^^2^ Although there are many clinical reports of outcomes in these high-risk patients, little is known on whether revascularization using these techniques can improve LVEF, a key surrogate of cardiac output and LV systolic function,^3^ and there remains a profound paucity of data on improvement in LVEF at midterm follow-up after HRPCI. Historically, there is a propensity for PCI outcomes reporting to focus more exclusively on the acute time period after PCI (<30 ​​days), with emphasis on major adverse cardiac and cerebrovascular events (MACCE).

The observational, multicenter RESTORE EF study was designed to address this question. The aim of the present study was to examine whether, in an ideal patient population with successful HRPCI using Impella hemodynamic support and without the need for subsequent procedures, there is an impact on LV function at 90-day follow-up. If midterm LVEF improvement exists in this carefully designed population, this positive signal could then be followed with more comprehensive studies or, conversely, indicate that this aggressive HRPCI approach does not have an impact on LV function.

## Methods

### Study design

**Figure 1:**
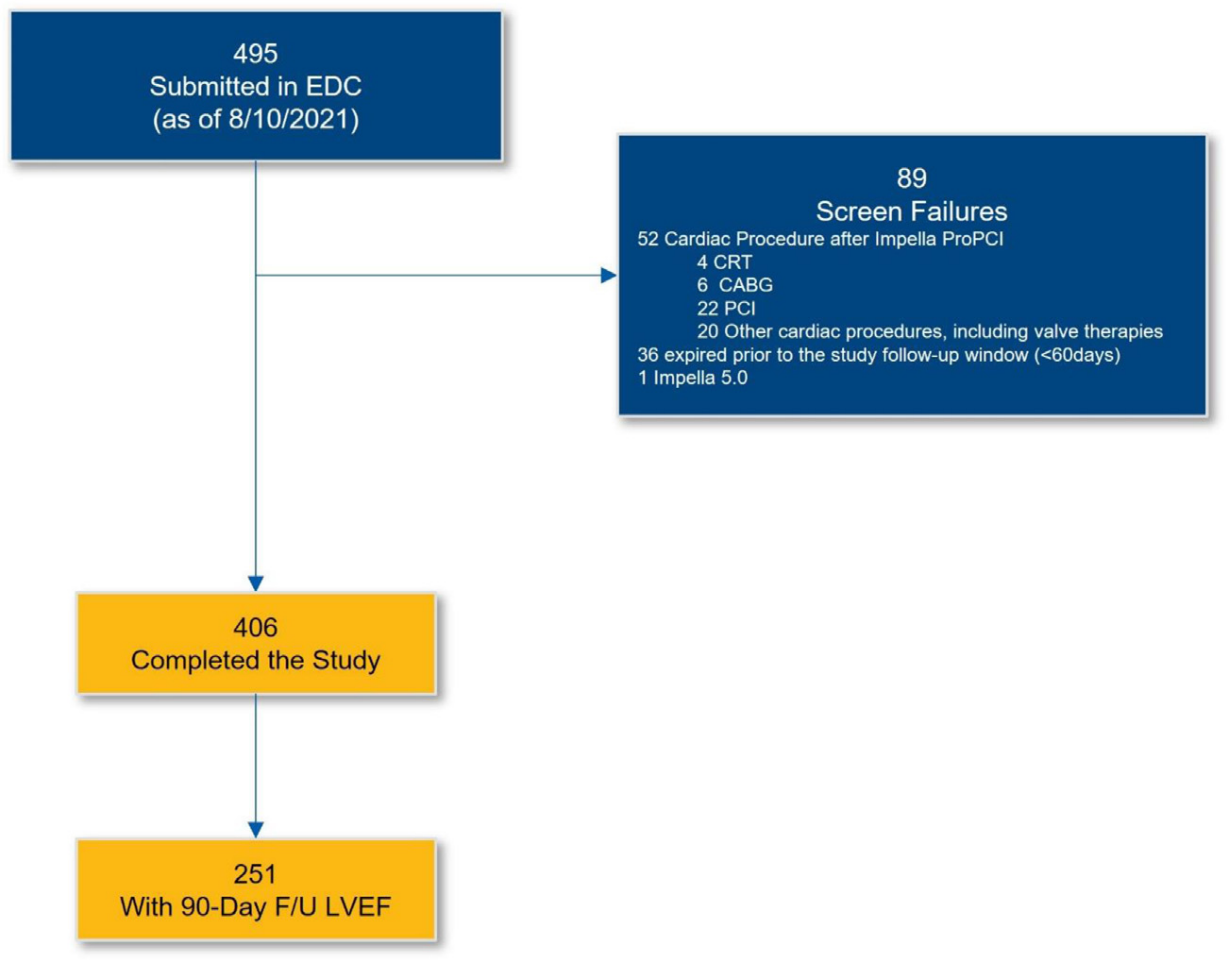
Patient flow chart. CABG, coronary artery bypass surgery; CRT, cardiac resynchronization therapy; EDC, electronic data capturing system; LVEF, left ventricular ejection fraction; PCI, percutaneous coronary intervention.

RESTORE EF (NCT04648306) is a multicenter, retrospective analysis of a prospectively collected observational data set that aimed to assess 90-day change in LVEF and clinical symptoms of heart failure and angina in a contemporary cohort of patients who received standard of care, percutaneous ventricular assist device-supported, elective or urgent PCI performed by the investigator at US centers identified for expertise in HRPCI and Impella utilization. Study operators were selected based on procedural volume and current implementation of contemporary practices such as safe vascular access techniques of large bore devices, complete revascularization, lesion preparation, intravascular imaging, and invasive hemodynamic monitoring in appropriately selected patients. All patients who underwent elective or urgent Impella-supported HRPCI were screened for study inclusion by the investigator. Patients were excluded under the following criteria: a) cardiac procedure after HRPCI (ie, CABG, additional PCI, cardiac resynchronization therapy, or other cardiac procedures including valve therapies) or b) patients had not reached the 90-day follow-up window (defined as 60-180 ​​days). Under these criteria, those patients who died before day 60 (the start of the 90-day follow-up window) were excluded (Figure 1) and not included in the population for analysis, comprising 36 patients out of 495 screened for study inclusion (7.3%). Patients who underwent Impella-supported HRPCI within 180 ​​days prior to site activation were also considered eligible for inclusion, if they met the study criteria and had a prospective LVEF assessment within the study follow-up window.

**Figure 2:**
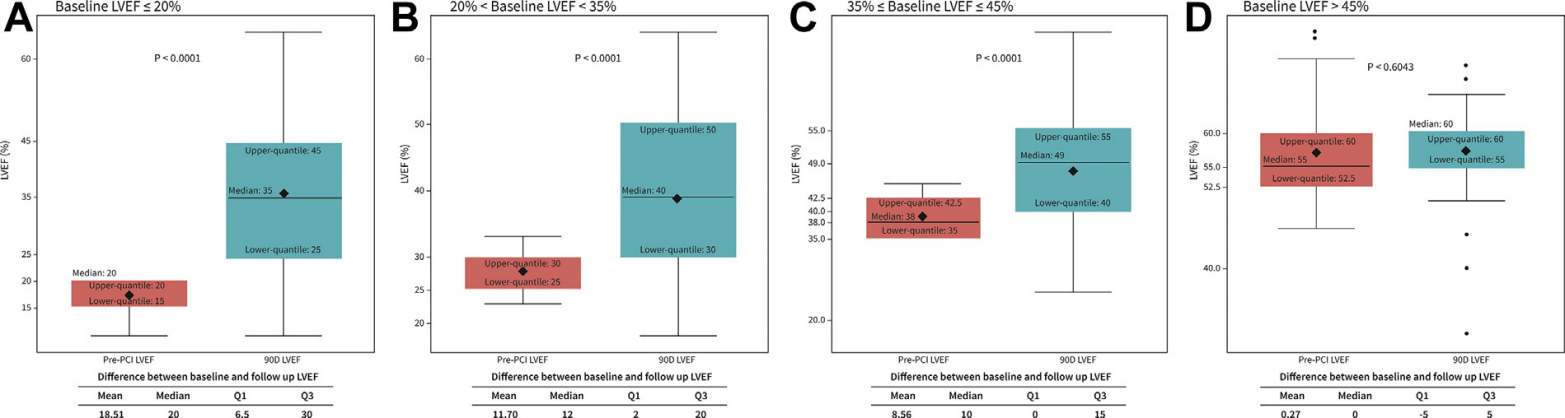
Follow-up LVEF improvement stratified by baseline LVEF quartile. Paired baseline and 90-day follow-up LVEF values were compared for each baseline LVEF quartile. Improvement in LVEF at 90 ​​days was significant for all quartiles, with the exception of patients with near-normal baseline LVEF (>45%). (**A**) Patients with baseline LVEF ≤20%. (**B**) Patients with baseline LVEF >20% and <35%. (**C**) Patient with LVEF ≥35% and ≤45%. (**D**) Patients with baseline LVEF >45%. LVEF, left ventricular ejection fraction. ∗The black squares represent the mean. The black dots represent possible outliers.

**Figure 3:**
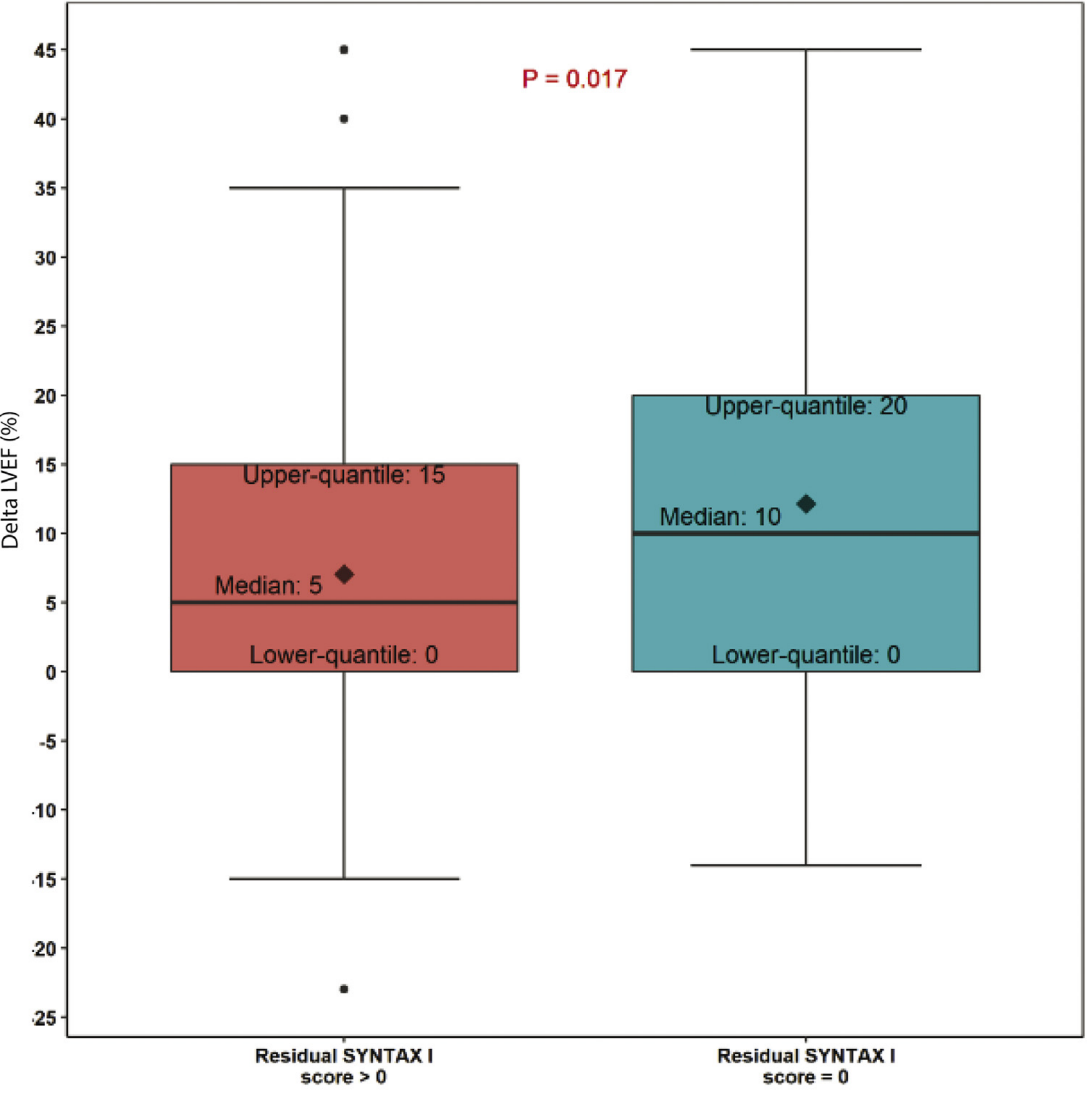
Extent of LVEF improvement at 90-day follow-up corelates with completeness of revascularization. Complete revascularization (defined as residual SYNTAX 1 score of 0; *N* = 91) was found to be associated with significantly greater 90-day LVEF improvement, compared with those patients with incomplete revascularization (residual SYNTAX I score > 0; *N* = 105), at a mean follow-up of 117 ​​± ​​38 ​​days (median, 104 ​​days). LVEF, left ventricular ejection fraction. The black squares represent the mean. The black dots represent possible outliers.

**Figure 4:**
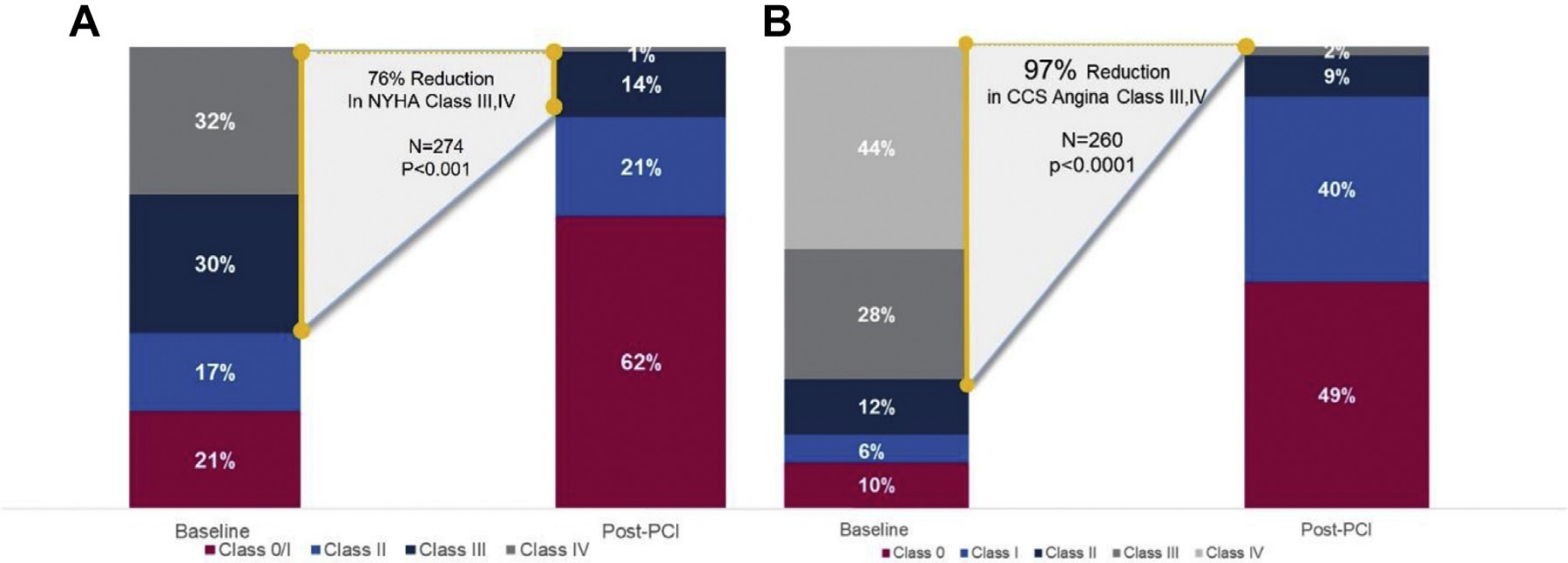
Heart failure and anginal symptom improvement at the last follow-up. (**A**) At a mean follow-up of 108 ​​± ​​70 ​​days (median, 108 ​​days), 15% of patients were classified as NYHA class III/IV compared to 62% at baseline assessment. (**B**) At a mean follow-up of 110 ​​± ​​77 ​​days (median, 101 ​​days), 2% of patients were classed as CCS Angina class III/IV (0% class IV, 2% class III) compared to 72% at baseline assessment. CCS, Canadian Cardiovascular Society; NYHA, New York Heart Association; PCI, percutaneous coronary intervention.

A central institutional review board and/or local institutional review board provided approval for the study protocol at all sites. Requirement for informed consent was waived for all patients due to the retrospective nature of the analysis, the on-label, observational study design, and more importantly absence of personal health care information data collection. All patients received either the Impella 2.5 or CP device, per discretion of the treating physician, with Impella support initiated prophylactically prior to initiation of HRPCI.

### Study endpoints

The primary endpoint was assessment of LVEF at 90 ​​days after PCI (60- to 180-day window). Follow-up echocardiographic LVEF assessment at 90 ​​days was per routine clinical course and was subject to physician discretion, due to the observational and noninterventional nature of this study (ie, physicians may not have opted for follow-up LVEF assessment in patients with normal or near-normal baseline LVEF, or due to curtailment of nonurgent services during the COVID-19 pandemic, which had significant overlap with the study time period). Secondary endpoints included change in heart failure and angina symptoms at follow-up assessment, as well as completeness of revascularization. Classification of heart failure and angina severity at baseline and post-PCI follow-up were per the New York Heart Association (NYHA) functional classification and Canadian Cardiovascular Society (CCS) angina grade, respectively. Qualitative physician assessment of symptoms and well-being improvement at follow-up was also recorded. SYNTAX score I and II were obtained before PCI. Completeness of revascularization was determined by residual SYNTAX score I, with a residual SYNTAX score of 0 defined as complete revascularization.^4^ Local site principal investigators assessed pre- and post-PCI SYNTAX scores and LVEFs.

### Statistical analysis

Baseline and 90-day LVEF measurements were compared using paired *t* test comparison. Categorical and continuous datapoint comparisons were performed using χ^2^ analysis and unpaired *t* test comparisons, respectively. A *P* value ​​< ​​.05 was considered significant.

## Results

### Patient and procedural characteristics

Four hundred and six subjects treated by 23 operators at 22 US sites were enrolled from August 2019 to May 2021 (Figure 1). Mean patient age was 70.2 ​​± ​​11.4 ​​years; 106 patients (26.1%) were female. All collected baseline characteristics are reported in Table 1. Baseline LVEF was 37 ​​± ​​16%, with 224 patients (77.0%) being surgical turndowns. The baseline SYNTAX score I was 29.7 ​​± ​​14.0; baseline SYNTAX score II was 52.6 ​​± ​​15.0. An average of 2.9 ​​± ​​1.1 lesions were treated, with 52.3% of patients undergoing atherectomy (Table 2). Vascular complications requiring blood transfusion occurred in 2.5% of patients.

**Table 1:** Baseline characteristics, all patients.

	(*N* ​​= ​​406)
Age, y	
Mean ​​± ​​SD, *N*	70.2 ​​± ​​11.4 (406)
Median (IQR)	71 (60.8-78.3)
Female	26.1% (106/406)
Pre-PCI LVEF, %	
Mean ​​± ​​SD, *N*	37 ​​± ​​16 (406)
Median (IQR)	35 (25-50)
NYHA class III/IV	60.2% (209/347)
CCS Angina class III/IV	68.9% (244/354)
Pre-PCI SYNTAX I	
Mean ​​± ​​SD, *N*	29.7 ​​± ​​14 (308)
Median (IQR)	28 (20-36)
Pre-PCI SYNTAX II	
Mean ​​± ​​SD, *N*	52.6 ​​± ​​15.0 (279)
Median (IQR)	53 (42-64)
Surgical turndown	77.0% (224/291)
Creatinine pre-Impella	
Mean ​​± ​​SD, *N*	1.6 ​​± ​​1.7 (405)
Median (IQR)	1.1 (0.9-1.4)
No. diseased vessels	
1	11.9% (45/379)
2	54.9% (208/379)
3	33.2% (126/379)
No. significant lesions (≥50% stenosis)	
Mean ​​± ​​SD, *N*	2.9 ​​± ​​1.4 (406)
Median (IQR)	3 (2-4)
Viability study performed	11.7% (41/351)
Viable myocardium	87.8% (36/41)

Continuous data are reported as both mean ​​± ​​SD and median (IQR); categorical data are reported as percentage (numerator/denominator).

CCS, Canadian Cardiovascular Society; IQR, interquartile range; LVEF, left ventricular ejection fraction; NYHA, New York Heart Association; PCI, percutaneous coronary intervention.

**Table 2:** Procedural characteristics, all patients.

	(*N* ​​= ​​406)
Impella device used	
Impella 2.5	26.4% (107/406)
Impella CP	73.6% (299/406)
Elective PCI	73.2% (292/399)
Urgent PCI	26.8% (107/399)
No. lesions treated	
Mean ​​± ​​SD, *N*	2.9 ​​± ​​1.1 (406)
Median (IQR)	3 (2-4)
Atherectomy	52.3% (180/344)
Duration of support, h	
Mean ​​± ​​SD, *N*	3.9 ​​± ​​10.8 (406)
Median (IQR)	2.0 (1.0-2.0)
Residual SYNTAX I	
Mean ​​± ​​SD, *N*	5.4 ​​± ​​10.4 (286)
Median (IQR)	2 (0-7.3)

Continuous data are reported as both mean ​​± ​​SD and median (IQR); categorical data are reported as percentage (numerator/denominator).

IQR, interquartile range; PCI, percutaneous coronary intervention.

### Follow-up outcomes

Through 29 ​​days, 10.5% of patients were readmitted to the hospital (Table 3). In those patients included in the final study population (ie, survived to the start of the 90-day follow-up window), survival through average last follow-up of 186 ​​± ​​86 ​​days was 97.3% (see Table 3 for mortality in all screened patients [*N* = 495] at time of support, 30, 90, and 180 ​​days, with cumulative mortality of 9.5% in all screened patients through the end of study follow-up). Per clinician assessment, overall symptom and well-being improvement was observed in 91.2% of patients at the last follow-up.

**Table 3:** Clinical outcome at 90-day follow-up, all patients.

	(*N* ​​= ​​406)
Vascular complications requiring blood transfusion	2.5% (10/406)
29-d readmission	10.8% (37/342)
Overall symptom and well-being improvement	91.2% (313/343)^a^
Mortality during follow-up for all subjects enrolled^b^	2.7% (11/406)
Mortality for all subjects screened (*N* ​​= ​​495)^c^	
Mortality on support	0.6% (3/495)
Mortality at 29 ​​d (cumulative)	6.9% (34/495)
Mortality at 90 ​​d (cumulative)	8.1% (40/495)
Mortality at 180 ​​d (cumulative)	9.5% (47/495)

Categorical data are reported as percentage (numerator/denominator).

a Overall symptom and well-being improvement comprises subjective physician assessment of patient condition at the 90-day follow-up visit.

b Mortality reported is only comprehensive of those patients who were enrolled in the study. Those patients who did not return for follow-up, in the 90-day visit window (60-180 days), were not included in the study population; mortality therefore reflects deaths that occurred after patient returned for follow-up, assessment (ie, is included in the study analysis).

c Of 495 patients screened for study eligibility, a total of 36 subjects screened for study eligibility expired prior to the start of the primary endpoint follow-up window (60 ​​days) and were therefore excluded from enrollment (Figure 1). From 60 ​​days through the end of the study follow-up window (180 ​​days), 11 patients (2.7%) enrolled in the study expired.

### Change in LVEF

In 251 patients with both baseline and 90-day follow-up LVEF measurements available, baseline LVEF improved from 35 ​​± ​​15% to 45 ​​± ​​14% at 90 ​​days (*P* < .0001; Central Illustration).

**Central Illustration:**
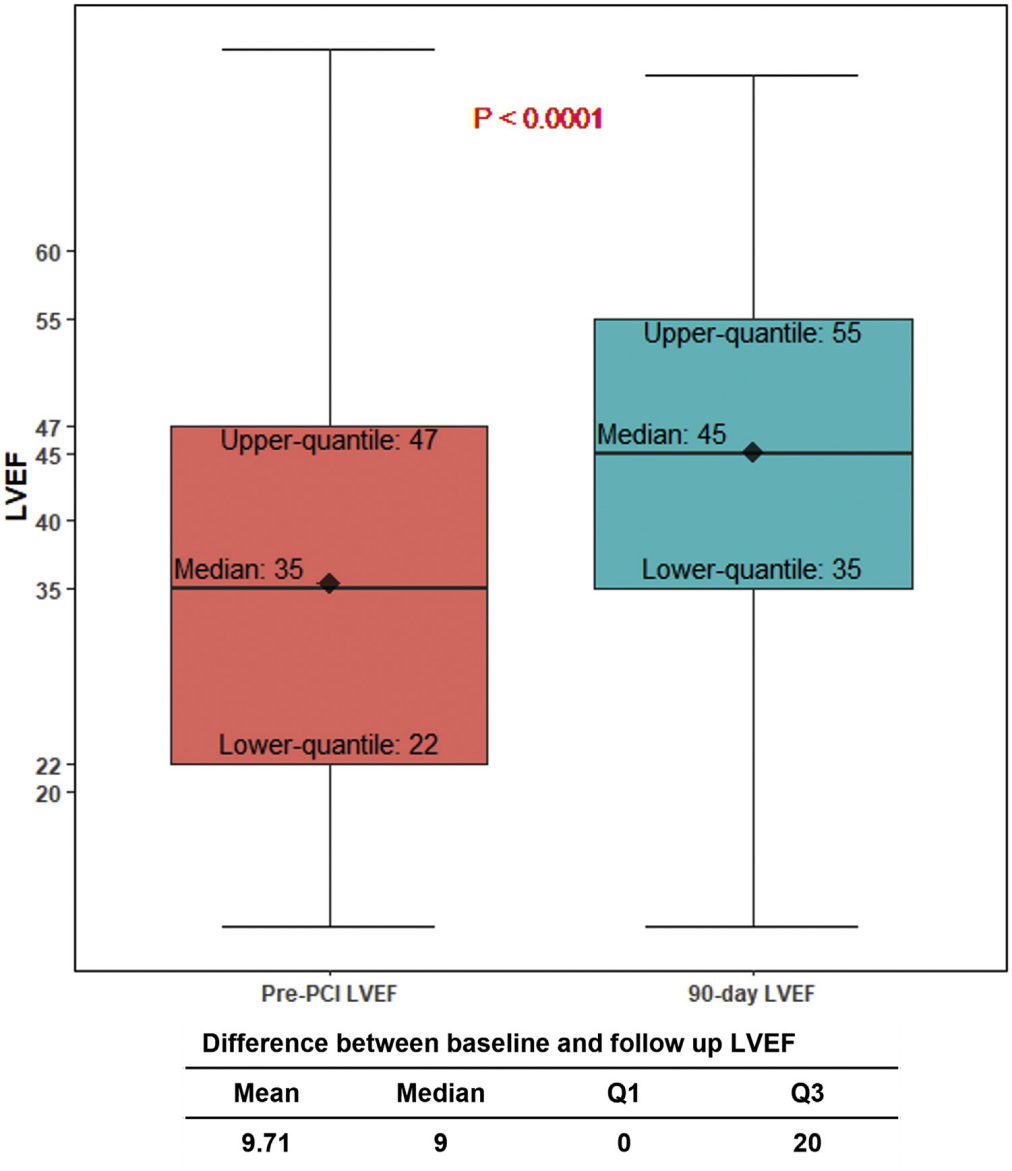
LVEF improvement at 90-day follow-up after Impella-supported HRPCI. Two hundred fifty-one patients had both available baseline and 90-day follow-up LVEF measurement, at a mean follow-up of 120 ​​± ​​40 ​​days (median, 108 ​​days). Fourteen had a follow-up LVEF measured at ≥180 ​​days after PCI. LVEF values are expressed as median (interquartile range) and mean ​​± ​​standard deviation. HRPCI, ​​high-risk percutaneous coronary intervention; LVEF, ​​left ventricular ejection fraction.

Improvement in LVEF was most pronounced in those with a baseline LVEF ≤20% (Figure 2). Subset analyses of LVEF improvement across baseline LVEF quartiles found that all quartiles showed significant improvement at 90 ​​days with the exception of patients with baseline LVEF >45%, who did not show significant improvement in LVEF, but nonetheless experienced significant improvement of NYHA class III/IV heart failure symptoms and CCS class III/IV anginal symptoms at follow-up assessment (Supplemental Fig. S1). Subset analyses of the impact of extent of revascularization on LVEF found that LVEF improvement at 90-day follow-up was significantly higher in subjects with residual SYNTAX Score I of 0 (10% LVEF improvement vs 5% improvement in those with residual SYNTAX Score I ​​> ​​0; *P* ​​= ​​.007; Figure 3).

### Change in clinical symptoms

In 274 patients with both baseline and follow-up NYHA Heart Failure classification, 62% of patients were classified as NYHA class III or IV at baseline assessment (32% class IV, 30% class III). At the last follow-up, percentage of patients classed as NYHA class III/IV decreased to 15% (*P* ​​< ​​.0001; Figure 4), comprising 1% class IV and 14% class III. In 260 patients with both baseline and follow-up CCS Angina classification, 72% were classified as CCS class III or IV at baseline assessment (44% class IV, 28% class III), decreasing to 2% at the last follow-up (*P* ​​< ​​.0001; Figure 4), comprising 0% class IV and 2% class III.

## Discussion

In this contemporary study of patients undergoing nonemergent HRPCI with an Impella support, we observed a significant improvement in LVEF at 90 ​​days in all patients except those with near-normal LVEF (>45%) at baseline. There was also a significant improvement in anginal CCS class and NYHA Heart Failure class, including those with near-normal baseline LVEF. Of note, patients who had more complete revascularization, characterized by a residual SYNTAX score of 0, had significantly greater improvement in LVEF at 90 ​​days. It should be noted, these findings were observed in an ideal patient population that underwent successful HRPCI with an Impella support, in which all patients survived to the 90-day follow-up window and had no intervening cardiac procedures after PCI. This was a critical part of the study design, in order to more confidently associate LVEF improvement with the index PCI procedure, as there were no additional procedures in the interim to serve as confounders. This analysis of an observational database represents a “best case” real-world scenario, with findings that are hypothesis-generating and suggest the value of supported HRPCI in this population, meriting further exploration in robustly designed trials and large, all-comer registries.

Patients with multivessel coronary artery disease (CAD) and concomitant ischemic cardiomyopathy with reduction in LVEF represent a challenging cohort of patients to treat. By guidelines, CABG is served a class IIa indication for improvement in survival in patients with moderately reduced LVEF (35%-50%) and presence of viable myocardium and a class IIb indication for severely reduced LVEF (<35%) regardless of viability.^5^ Given the increasing age and comorbidities among patients encountered in contemporary clinical practice,^6^ a significant number of these patients are either at high risk or ineligible for surgical revascularization.^7^^,^^8^ Following a heart team discussion or due to patient preference, hemodynamically supported PCI may be a viable alternative, if not the only option for revascularization, in these patients. In our current study, 77.0% of patients were surgical turndowns. It is important to note that historically, this patient population characterized by surgical ineligibility and depressed LVEF has been excluded from conventional CABG vs PCI trials.^9^, ^10^, ^11^, ^12^, ^13^

The PROTECT II trial was a RCT of nonemergent HRPCI patients, which demonstrated that LV support with Impella 2.5 during HRPCI was associated with a lower major adverse event rate than intra-aortic balloon pump at 90 ​​days.^14^ Prospective, observational data from the contemporary PROTECT III study have recently demonstrated a reduction of composite MACCEs with utilization of Impella 2.5 or CP when compared to rates reported with the Impella 2.5 in the earlier PROTECT II trial despite these patients having more complex CAD.^15^ This has been hypothesized to be secondary to contemporary best practices including patient selection and techniques for HRPCI (ie, more extensive revascularization and higher use of atherectomy, wider utilization of vascular closure devices). Recently, Russo et al^16^ analyzed data from PROTECT II and the catheter-based Ventricular Assist Device (cVAD) registry to evaluate improvement in LVEF following Impella-supported HRPCI. The study only included patients with paired echocardiographic data at baseline and at least 30 ​​days of follow-up. Among the 689 patients included in the study, there was an improvement in the mean LVEF from 24.8 ​​± ​​9.9% to 31.4 ​​± ​​13.3% after PCI (*P* ​​< ​​.001). In our current study, we found that at 90-day follow-up, there was an improvement in LVEF from 35 ​​± ​​15% to 45 ​​± ​​14% following Impella-supported HRPCI. We further found that the extent of complete revascularization characterized by residual SYNTAX score correlated with the degree of LVEF improvement.

As previously mentioned, there is a growing body of evidence demonstrating that complete revascularization (as opposed to culprit-lesion-only revascularization) is associated with improved survival and reduced rate of long-term major adverse cardiac events and repeat revascularization. To date, this has been reported more commonly in the setting of ST-elevation myocardial infarction^17^, ^18^, ^19^ although recent large, observational studies reported by Rathod et al and Kim et al show similar findings in non−ST-elevation myocardial infarction patient populations.^20^^,^^21^ In the setting of acute myocardial infarction complicated by cardiogenic shock, evidence points to similar early outcomes after PCI regardless of whether a culprit-lesion-only or complete revascularization strategy is pursued^22^, ^23^, ^24^ although whether these strategies yield different long-term outcomes has not been sufficiently explored.

In a subanalysis of the SYNTAX trial that aimed to validate the residual SYNTAX score as a predictor of long-term mortality, Farooq et al^4^ found a significant association with increasing residual SYNTAX Score and 5-year mortality. Those with residual scores from 0 (indicating complete revascularization) to 8 were found to have similar long-term mortality (8.5%-11.4%). However, those with a SYNTAX score >8 were associated with 35.3% mortality at 5 ​​years (*P* ​​< ​​.001). An analysis of New York State’s PCI data also found significantly higher risk of mortality in patients with incomplete revascularization although this varied considerably depending on the degree of incomplete revascularization and whether the incompletely revascularized vessels were totally occluded.^25^ Jimenez-Navarro et al^26^ found that survival benefit of complete revascularization was significantly more pronounced in patients with diabetes. These data indicate that the clinical benefit of complete revascularization may be largely individualized based on comorbidity burden and anatomical complexity. Those patients at higher risk during PCI procedures may be more likely to benefit from complete revascularization, with analyses from the Single-Staged Compared With Multi-Staged PCI in Multivessel NSTEMI Patients and SYNTAX trials indicating that complete revascularization achieved in a single stage (as opposed to multistage) was associated with lower MACCE through hospital discharge and 5 ​​years, respectively, although there are confounding variables such as comorbid conditions to consider in these analyses. Furthermore, the Single-Staged Compared With Multi-Staged PCI in Multivessel NSTEMI Patients trial excluded those patients with a SYNTAX score I ​​>32, whereas in the present study, 32.1% of patients had a pre-PCI SYNTAX score I ​​>32.^27^^,^^28^

In recently presented findings from the multicenter Outcomes of Surgically Ineligible Patients With Multivessel CAD (OPTIMUM) registry, which reported clinical outcomes in surgically ineligible patients undergoing PCI, Kandzari et al^29^ reported a 30-day mortality rate lower than surgeon-predicted mortality, at 5.6%. However, at 6 ​​months, mortality had more than doubled to 12.3%, underscoring the significant disease burden and high-risk nature of this patient population. In our study, which is also largely comprised of surgical turndown patients (77%), we report a higher 30-day mortality rate of 6.9% (in all screened patients), increasing to 9.5% at 180 ​​​days. It is important to note that patients in the OPTIMUM trial had a baseline SYNTAX score of 32.4, decreasing to a residual score of 15 after PCI. In our study, the mean baseline SYNTAX score of 29.7 decreased to 5.4 after PCI. In a subanalysis, the OPTIMUM investigators found that those patients with a residual score <8, indicating more complete revascularization, had a lower 30-day mortality rate, although this did not attain significance (3.9% vs 6.3%; *P* = .19).^29^ We eagerly await full publication to learn whether they identify any correlation with complete revascularization and midterm mortality.

In the Roma-Verona registry, Burzotta et al^19^ found that a greater extent of revascularization with Impella-supported HRPCI (assessed with British Cardiovascular Intervention Society myocardial jeopardy score revascularization index ≥0.8) was found to be significantly protective for later mortality; in fact, it was 1 of only 2 significant predictors identified for mortality (the other being EuroSCORE I ​​> ​​11) although residual SYNTAX score was not found to be similarly predictive. Mean pre- and post-PCI SYNTAX scores in this study were 31 ​​± ​​10 and 12 ​​± ​​8.7, respectively. Furthermore, the authors found that completeness of revascularization had a significant association with LVEF improvement through 14-month follow-up (*P* ​​= ​​.002). To our knowledge, this is the only previously published data on longer term follow-up LVEF after HRPCI, as well as the only published findings connecting complete revascularization with greater LVEF improvement, although it is limited by a relatively small patient population (*N* = 86) at 2 Italian centers. There remains a lack of quantitative data on midterm LVEF improvement following HRPCI and its association, or lack thereof, with degree of revascularization achieved in HRPCI—this constitutes 1 of the more novel findings of this analysis.

Given that the vast majority of patients in our study were surgical turndowns (79.5%) with multivessel CAD, the intent of the operators was to perform as complete revascularization as feasible in a single setting with hemodynamic support. These data support a strategy aimed at complete revascularization in this high-risk cohort contributed to the change in LVEF, a surrogate endpoint, as well as anginal symptoms and heart failure classifications. We propose that this practice of complete revascularization contributed significantly to not just an improvement in LVEF but also a marked improvement in angina and heart failure functional class. It is to be noted that even in the subgroup of patients with only borderline depressed LVEF, the procedure had a significant benefit in terms of improvement in angina and heart failure classifications.

## Limitations

The primary limitation of our study is the observational, nonrandomized nature of the study design and the lack of a comparator. Additionally, we relied on investigator-reported data, and there was no angiographic or echocardiographic core lab, or independent clinical events committee adjudication. By design, the final study population comprised only those who survived to the 90-day follow-up window, with no intervening cardiac procedures. A significant number of patients (61.8%) who met these inclusion criteria and were included in the final study population did not, in fact, undergo 90-day follow-up LVEF assessment. In some cases, this was due to patients missing their appointment, and in others, it was due to physician opting not to order 90-day LVEF assessment in subjects with normal or near-normal baseline LVEF. As this was an observational study, 90-day LVEF assessment was strictly per physician discretion. It should be noted that most of the study was conducted during the COVID-19 pandemic, during which time many centers experienced a cessation of nonurgent services in hospitals, impairing the ability to obtain follow-up echocardiograms in some cases. Furthermore, although we intentionally excluded patients who received subsequent cardiac procedures that may potentially contribute to improvement in LVEF and confound analysis of study endpoints (such as valve therapies, cardiac resynchronization therapy, subsequent CABG, or staged PCI), the role that optimal heart failure medical therapy played in augmenting LV function cannot be reliably discerned, as data on these medications were not collected in the study.

## Conclusions

This observational study suggests that hemodynamically supported HRPCI may afford a significant improvement in LVEF at 90 ​​days, along with significant relief of angina and heart failure symptoms, in an ideal patient population with successful HRPCI and without the need for subsequent procedures. In this setting, complete revascularization was associated with a more significant improvement in LVEF. Those patients with normal baseline LVEF (>45%) did not show significant improvement in LVEF although they did exhibit significant improvement in heart failure and angina symptoms at 90 ​​days. These hypothesis-generating findings merit further assessment in more comprehensive studies.

## Declaration of competing interest

Dr Morris and Dr Wollmuth are consultants and speakers for Abiomed. Dr Patel is a consultant for Abbott, Medtronic, Terumo, and Chiesi. Dr Dahle is a consultant and proctor for Abiomed. Dr Bharadwaj is a consultant and speaker for Abiomed and CSI. Dr Waggoner is a consultant for Abiomed, Abbott, Acutus, Boston Scientific, Edwards, and Medtronic. Dr Mahmud is a consultant for Abiomed. All other authors have no relationships with industry to disclose; specifically, they do not have any equity or financial involvement, nor are they paid consultants to Abiomed.
