# Supplementary material for: Ejection Fraction Improvement Following Contemporary High-Risk Percutaneous Coronary Intervention: RESTORE EF Study Results
Source: J Soc Cardiovasc Angiogr Interv. 2022 Aug 13;1(5):100350. doi: 10.1016/j.jscai.2022.100350 (PMC11307872; doi:10.1016/j.jscai.2022.100350)
Supplement: Supplemental Fig. S1 [file mmc1.docx]

# ­­­­­Supplementary Figures


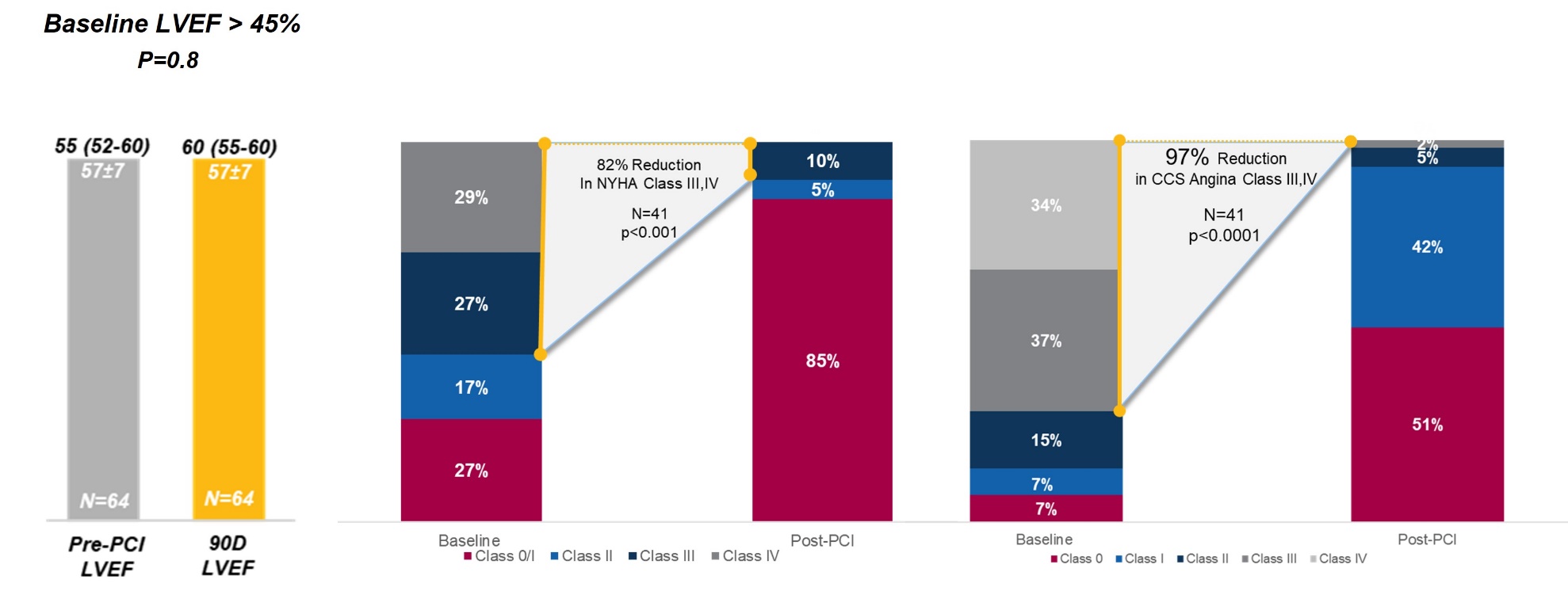


**Supplementary Figure 1. Heart Failure and Anginal Symptom Improvement in Patients with Near-Normal Baseline LVEF (> 45%). A)** Though no significant LVEF improvement was observed in 64 patients with baseline LVEF > 45%, **B)** at a mean follow-up of 119 ± 93 days (median, 102 days), 10% of patients with baseline LVEF > 45% were classified as NYHA Class III/IV (0% Class IV, 10% Class III) compared to 56% at baseline assessment. **C)** At a mean follow-up of 138 ± 114 days (median, 1015 days), 7% were classified as CCS Angina Class III/IV (2% Class IV, 5% Class III) compared to 71% at baseline assessment.
